# Choosing the best heuristic for seeded alignment of DNA sequences

**DOI:** 10.1186/1471-2105-7-133

**Published:** 2006-03-13

**Authors:** Yanni Sun, Jeremy Buhler

**Affiliations:** 1 Department of Computer Science and Engineering, Washington University, St. Louis, MO, USA

## Abstract

**Background:**

Seeded alignment is an important component of algorithms for fast, large-scale DNA similarity search. A good seed matching heuristic can reduce the execution time of genomic-scale sequence comparison without degrading sensitivity. Recently, many types of seed have been proposed to improve on the performance of traditional contiguous seeds as used in, e.g., NCBI BLASTN. Choosing among these seed types, particularly those that use information besides the presence or absence of matching residue pairs, requires practical guidance based on a rigorous comparison, including assessment of sensitivity, specificity, and computational efficiency. This work performs such a comparison, focusing on alignments in DNA outside widely studied coding regions.

**Results:**

We compare seeds of several types, including those allowing transition mutations rather than matches at fixed positions, those allowing transitions at arbitrary positions ("BLASTZ" seeds), and those using a more general scoring matrix. For each seed type, we use an extended version of our Mandala seed design software to choose seeds with optimized sensitivity for various levels of specificity. Our results show that, on a test set biased toward alignments of noncoding DNA, transition information significantly improves seed performance, while finer distinctions between different types of mismatches do not. BLASTZ seeds perform especially well. These results depend on properties of our test set that are not shared by EST-based test sets with a strong bias toward coding DNA.

**Conclusion:**

Practical seed design requires careful attention to the properties of the alignments being sought. For noncoding DNA sequences, seeds that use transition information, especially BLASTZ-style seeds, are particularly useful. The Mandala seed design software can be found at .

## Background

### Seed design for DNA sequence alignment

Comparing nucleic acid sequences is one of the most important tasks in computational biology. Although many sequence alignment algorithms have been designed, the rapid increase in size of genome databases continues to present alignment algorithms with the challenge of finding good alignments (i.e. those with high scores) efficiently.

Seeded alignment is the dominant technique for large-scale genomic sequence comparisons, and BLASTN [1] is the most popular implementation of it. In BLASTN, exact matches of *w *contiguous residues between sequences are identified and then extended into alignments by dynamic programming. More recent work extends BLASTN's contiguous match heuristic to find more general patterns of conservation, which are commonly called *seeds*. Seeds have been used not only for large-scale local alignment but also as anchor points in whole-genome and multiple sequence alignment algorithms [2,3].

It has been shown [4] that seed design is important to the sensitivity and specificity of seeded alignment. To improve on traditional contiguous seeds used in, e.g., BLASTN [1], *spaced seeds *were proposed, initially as part of the FLASH indexed search tool [5]. A spaced seed spanning *s *bases requires matching bases at only a subset of the positions {0,1, ..., *s *- 1}. PatternHunter [4] used a carefully designed spaced seed based on a simple i.i.d. alignment model to increase sensitivity. Mandala [6] built a DFA to evaluate the sensitivity of spaced seeds under a κth-order Markov model; a more accurate hidden Markov model was used in [7]. Algorithms for multi-seed design are described in [8-11]. All of the seeds mentioned above distinguish only between matched and mismatched residue pairs. In this paper, we call them *basic seeds*.

The BLASTZ alignment program [12] adopted the optimal spaced seed designed by PatternHunter. In order to increase sensitivity, they allowed a transition mutation (A-G, G-A, C-T, or T-C) at any one of the inspected positions in PatternHunter's seed. We generalize this definition to arbitrary seeds allowing transition mutations at fixed positions, which we call *transition seeds*.

In BLASTP [1], a protein alignment contains a seed match if the sum of scores of three consecutive amino acid pairs starting from some offset in the alignment exceeds a specified threshold. The individual score of each residue pair comes from a scoring matrix *M *modeling the rate of evolutionary change. We apply BLASTP-like seeds to DNA sequence comparison by computing the sum of the scores in the positions inspected by a basic seed. These seeds are called *score seeds*. Score seeds can also implement the inexact seeds used in BLAT [13] and CHAOS [3].

Score seeds are a special type of vector seed [14] using only binary vectors. [14] observed that most vector seeds that empirically worked well had binary seed vectors. Thus, we do not focus on a general vector seed's performance in this work. We note that transition seeds could also be represented by vector seeds with a carefully designed seed vector and threshold.

### Motivation for our work

Although none of the above seed types is novel, there is an absence of practical guidance to picking good seeds due to a lack of detailed performance comparison among basic, transition, and score seeds. The purpose of this work is to compare the above-mentioned seed types in a common experimental framework, and to derive practical guidance for choosing an appropriate seed heuristic for seeded alignment in genomic DNA.

A comparison of seeds must consider both sensitivity to biologically meaningful alignments and specificity, which affects the computational cost of using a seed. Less stringent seeds, e.g. seeds that allow transition mutations as well as matches, are likely to yield better sensitivity, but at a significant cost in specificity. Although some recent works on multiple simultaneous seed design [8-11] have been aware of this tradeoff, they do not perform systematic evaluations to judge whether less stringent seeds confer a net benefit to seeded alignment, that is, whether they are consistently more sensitive for given specificity. This work summarizes both sensitivity and specificity of seeds using receiver operating characterstic (ROC) curves. We also consider, where appropriate, other issues affecting the computational cost of seeds.

As part of our work, we must *design *seeds; that is, we must choose particular seeds or sets of seeds that maximize performance among many seeds or sets of the same type. Seed design requires efficient computation of seed sensitivity and, at times, specificity. Methods for efficiently computing the sensitivity of a basic seed have been reported in several papers [4,6,7]. An efficient algorithm was used to compute sensitivity of score seeds in [14]. YASS [15] computed the sensitivity of a single transition seed. In this work, we extend our Mandala seed design software [6] to compute the sensitivities of transition seeds and score seeds. We also develop methods to estimate a seed's specificity where direct measurement of this quantity is computationally prohibitive. Previous work [4,14] computed seed specificity based on a simple i.i.d. background sequence model. To ensure a biologically more appropriate comparison, we compute specificity based on a more informative Markov sequence model.

To evaluate a seed's sensitivity, we use a set of alignments representing the type of sequence similarity to be found. While many previous papers [7,10,14] have focused on designing seeds for coding DNA regions, we are also interested in designing seeds for noncoding DNA alignment because comparative analysis between noncoding regions discloses important information about genome evolution and functional features [12,16] and is the basis of modern whole-genome alignment tools [17,18]. We therefore focus our study on seed performance in alignments biased strongly toward noncoding DNA, though we also consider the extent to which results in these alignments generalize to a more traditional coding-heavy test set.

The remainder of this work is organized as follows. After describing our methods, we present experimental results on the relative sensitivities and computational costs of different types of seed. We conclude by identifying lessons drawn from our experiments and indicate directions for future work.

### Formal definitions of seeds

A *basic seed π *is defined to be an ordered list of indices {*x*_1 _... *x*_*w*_} with *x*_1 _= 0. Two sequences *S *and *T *exhibit a *seed match *at offsets *i *and *j *if, for 1 ≤ *k *≤ *w*, *S*[*i *+ *x*_*k*_] = *T*[*j *+ *x*_*k*_]. The number of inspected positions *w *is the *weight *of *π*, while the distance *s *= *x*_*w *_+ 1 is its *span*. For a position *j *with 0 ≤ *j *≤ *s*, if *j * {*x*_1 _... *x*_*w*_}, then *y*_*i *_is a "don't care position" that could be a match or mismatch.

A *transition seed π*_*z *_with span *s *is defined to be a pair of ordered lists of distinct indices: (*X *= {*x*_1 _... *x*_*w*_}, *Z *= {*z*_1 _... *z*_*m*_}) with *x*_1 _= 0 or *z*_1 _= 0, *w *≤ *s *and *m *≤ *s*. Two sequences *S *and *T *exhibit a *transition seed match *at offsets *i *and *j *if two conditions are satisfied: 1) For 1 ≤ *k *≤ *w*, *S*[*i *+ *x*_*k*_] = *T*[*j *+ *x*_*k*_]; 2) For 1 ≤ *k *≤ *m*, either *S*[*i *+ *z*_*k*_] = *T*[*j *+ *z*_*k*_], or the two residues are both purines or both pyrimidines. We call each *x*_*i *_a *match position *and each *z*_*i *_a *transition position*. For any position *y*_*i*_, if *y*_*i *_<*s*, *y*_*i *_ *X *and *y*_*i *_ *Z*, it is a "don't care position" that could be a match, a transition, or a transversion. The number of inspected match positions *w *is the *match weight *of *π*_*z*_. The number of transition positions *m *is the *transition weight *of *π*_*z*_. The span of a transition seed is *max*(*x*_*w*_, *z*_*m*_) + 1.

A *score seed *is defined by a pair <*π*, *T*>, where *π *is a basic seed and *T *is a score threshold. Let *π *= {*x*_1 _... *x*_*w*_} with *x*_1 _= 0. Two sequences *S *and *T *exhibit a *score seed match *at offsets *i *and *j *if, for 1 ≤ *k *≤ *w*, ∑_*k *_*C*_*i*_+*x*_*k*_,*j*+*x*_*k *_≥ *T*. Here, *C*_*i*_+*x*_*k*_,*j*+*x*_*k *_is the score for the residue pair (*S*[*i *+ *x*_*k*_], *T*[*j *+ *x*_*k*_]). The individual score of every pair comes from a scoring matrix *M*. The weight *w *and span *s *of a score seed are defined by its underlying basic seed *π*.

A set of multiple simultaneous seeds Π = {*π*_1 _... *π*_*n*_} contains *n *seeds of the same type. A seed set Π matches an alignment if *at least one component seed *from Π matches this alignment. Based on this definition, the transition seed used in BLASTZ alignment [12] is actually a set of 12 transition seeds, each with *w *= 11 and *m *= 1. The seeds differ from each other in only one position. We call this set a *BLASTZ seed*, which is a special case of multiple transition seeds. Because multiple score seed design is a somewhat different problem from regular seed design [19], we will focus only on sets of transition seeds.

## Methods

### Seed sensitivity and specificity

A seed's *sensitivity *is the probability that a biologically meaningful alignment contains a match to the seed. In our experiments, a seed's sensitivity is measured by the fraction of a set of known alignments that contain the match to a specified seed.

Because seed design requires evaluating the sensitivities of hundreds or thousands of candidate seeds, we need a more efficient way to estimate sensitivity in order to search for optimal seeds or seed sets. The Mandala seed design software [6] contains efficient estimation procedures for basic seeds. For this work, we have extended Mandala to compute the sensitivity of a seed in a probabilistic alignment model ℳ
 MathType@MTEF@5@5@+=feaafiart1ev1aaatCvAUfeBSjuyZL2yd9gzLbvyNv2CaerbwvMCKfMBHbqedmvETj2BSbWenfgDOvwBHrxAJfwnHbqeg0uy0HwzTfgDPnwy1aqee0evGueE0jxyaibaieYdOi=BH8vipeYdI8qiW7rqqrFfpeea0xe9Lq=Jc9vqaqpepm0xbbG8FasPYRqj0=yi0lXdbba9pGe9qqFf0dXdHuk9fr=xfr=xfrpiWZqaaeaabiGaaiaacaqabeaadaqacqaaaOqaaGWaaiab=ntinbaa@3AA6@ that includes information other than simply the distribution of matches and mismatches. These extensions use Mandala's DFA-based sensitivity evaluator. For a transition seed, we create a three-symbol DFA whose size is at most *s*3^*s *- *w *- *m*^2^*m*^, where *s*, *w*, and *m *are the seed's span, match weight, and transition weight respectively. If the number of different values in a score matrix *M *is *D*, a *D*-symbol DFA can be built to evaluate the sensitivity of a score seed. When dynamic programming becomes expensive, i.e. for seeds with large span but small weight (e.g., *s *= 22, *w *= 9), we resort to Monte Carlo methods to estimate sensitivity, as described in [6].

The *false positive rate *(*fp rate*) of a seed, which is complementary to its *specificity*, is the probability that a seed match occurs at some position purely by chance. For single basic and transition seeds with moderate weights, we can efficiently compute fp rates directly by counting seed matches between pairs of unrelated sequences. This computation is effectively the same as using the seed to perform a similarity search, which can be done efficiently in practice. However, this direct counting approach becomes computationally quite expensive for sets of seeds, as we must make certain not to count hits to two seeds in a set as two distinct hits to the set.

Our current implementation of hit counting for score seeds is also computationally expensive, which poses a problem for designing such seeds. Setting a score seed's threshold *T *to obtain a target fp rate requires evaluation of multiple thresholds, and computing the fp rates for these thresholds by direct counting proved burdensome in practice. These considerations led us to seek more efficient, though slightly less accurate, computational strategies to estimate fp rates for score seeds and sets of transition seeds.

We estimate fp rates using a probabilistic model ℳ
 MathType@MTEF@5@5@+=feaafiart1ev1aaatCvAUfeBSjuyZL2yd9gzLbvyNv2CaerbwvMCKfMBHbqedmvETj2BSbWenfgDOvwBHrxAJfwnHbqeg0uy0HwzTfgDPnwy1aqee0evGueE0jxyaibaieYdOi=BH8vipeYdI8qiW7rqqrFfpeea0xe9Lq=Jc9vqaqpepm0xbbG8FasPYRqj0=yi0lXdbba9pGe9qqFf0dXdHuk9fr=xfr=xfrpiWZqaaeaabiGaaiaacaqabeaadaqacqaaaOqaaGWaaiab=ntinbaa@3AA6@_*b *_that characterizes alignments between a pair of unrelated, random sequences. Alignments in model ℳ
 MathType@MTEF@5@5@+=feaafiart1ev1aaatCvAUfeBSjuyZL2yd9gzLbvyNv2CaerbwvMCKfMBHbqedmvETj2BSbWenfgDOvwBHrxAJfwnHbqeg0uy0HwzTfgDPnwy1aqee0evGueE0jxyaibaieYdOi=BH8vipeYdI8qiW7rqqrFfpeea0xe9Lq=Jc9vqaqpepm0xbbG8FasPYRqj0=yi0lXdbba9pGe9qqFf0dXdHuk9fr=xfr=xfrpiWZqaaeaabiGaaiaacaqabeaadaqacqaaaOqaaGWaaiab=ntinbaa@3AA6@_*b *_arise from two independent sequences, each of which is modeled by a *k*th-order Markov process. The alignment model can be computed from the two underlying sequence models by multiplying their corresponding terms as follows:

Pr((*c*_1_,*c*_2_)|(*x*_1_,*y*_1_) ... (*x*_*k*_, *y*_*k*_))

= Pr(*c*_1_|*x*_1 _... *x*_*k*_) × Pr(*c*_2_|*y*_1 _... *y*_*k*_).

The next section shows how this model is used to estimate false positive rates.

### Computation of fp rates

Efficient computation of false positive rates, particularly for score seeds, is computationally nontrivial. Here, we describe a dynamic programming algorithm, inspired by Altschul's computation of sum score frequencies in NCBI BLAST [1], that computes fp rates for score seeds when ℳ
 MathType@MTEF@5@5@+=feaafiart1ev1aaatCvAUfeBSjuyZL2yd9gzLbvyNv2CaerbwvMCKfMBHbqedmvETj2BSbWenfgDOvwBHrxAJfwnHbqeg0uy0HwzTfgDPnwy1aqee0evGueE0jxyaibaieYdOi=BH8vipeYdI8qiW7rqqrFfpeea0xe9Lq=Jc9vqaqpepm0xbbG8FasPYRqj0=yi0lXdbba9pGe9qqFf0dXdHuk9fr=xfr=xfrpiWZqaaeaabiGaaiaacaqabeaadaqacqaaaOqaaGWaaiab=ntinbaa@3AA6@_*b *_takes the form of *k*th-order Markov model with *k *> 0. The same algorithm can be simplified to compute fp rates for other types of seed. We then extend our computation to deal with sets of seeds. Finally, we quantify how well our estimates of seeds' fp rates approximate the actual rates using a sample of randomly chosen seeds of several types.

#### Fp rate for a score seed

The fp rate of a score seed <*π*,*T*> is the probability that, at a fixed position in a pair of unrelated sequences, the total score of the positions (*x*_1_, ..., *x*_*w*_)inspected by *π *is at least *T*. Let A
 MathType@MTEF@5@5@+=feaafiart1ev1aaatCvAUfKttLearuWrP9MDH5MBPbIqV92AaeXatLxBI9gBamrtHrhAL1wy0L2yHvtyaeHbnfgDOvwBHrxAJfwnaebbnrfifHhDYfgasaacH8akY=wiFfYdH8Gipec8Eeeu0xXdbba9frFj0=OqFfea0dXdd9vqai=hGuQ8kuc9pgc9s8qqaq=dirpe0xb9q8qiLsFr0=vr0=vr0dc8meaabaqaciaacaGaaeqabaWaaeGaeaaakeaaimaacqWFaeFqaaa@3821@ be the sequence alphabet; alignments of unrelated sequences are described by a |A
 MathType@MTEF@5@5@+=feaafiart1ev1aaatCvAUfKttLearuWrP9MDH5MBPbIqV92AaeXatLxBI9gBamrtHrhAL1wy0L2yHvtyaeHbnfgDOvwBHrxAJfwnaebbnrfifHhDYfgasaacH8akY=wiFfYdH8Gipec8Eeeu0xXdbba9frFj0=OqFfea0dXdd9vqai=hGuQ8kuc9pgc9s8qqaq=dirpe0xb9q8qiLsFr0=vr0=vr0dc8meaabaqaciaacaGaaeqabaWaaeGaeaaakeaaimaacqWFaeFqaaa@3821@|^2^-symbol *k*th-order Markov model ℳ
 MathType@MTEF@5@5@+=feaafiart1ev1aaatCvAUfeBSjuyZL2yd9gzLbvyNv2CaerbwvMCKfMBHbqedmvETj2BSbWenfgDOvwBHrxAJfwnHbqeg0uy0HwzTfgDPnwy1aqee0evGueE0jxyaibaieYdOi=BH8vipeYdI8qiW7rqqrFfpeea0xe9Lq=Jc9vqaqpepm0xbbG8FasPYRqj0=yi0lXdbba9pGe9qqFf0dXdHuk9fr=xfr=xfrpiWZqaaeaabiGaaiaacaqabeaadaqacqaaaOqaaGWaaiab=ntinbaa@3AA6@_*b*_. For any residue pair *b *∈ A
 MathType@MTEF@5@5@+=feaafiart1ev1aaatCvAUfKttLearuWrP9MDH5MBPbIqV92AaeXatLxBI9gBamrtHrhAL1wy0L2yHvtyaeHbnfgDOvwBHrxAJfwnaebbnrfifHhDYfgasaacH8akY=wiFfYdH8Gipec8Eeeu0xXdbba9frFj0=OqFfea0dXdd9vqai=hGuQ8kuc9pgc9s8qqaq=dirpe0xb9q8qiLsFr0=vr0=vr0dc8meaabaqaciaacaGaaeqabaWaaeGaeaaakeaaimaacqWFaeFqaaa@3821@^2^, its score *M*[*b*] is given by a substitution matrix *M*.

The following algorithm computes the probability that, at any fixed offset in the alignment, the total score across all positions inspected by *π *is at least *T*. Let *δ *be a residue pair string of length *k*. For each position *j *from 0 to *s *(the span of *π*), define *P*_*j*_(*θ*, *δ*·*b*) to be the probability that the total score of the inspected positions up to *j *is *θ*, and that the last *k *+ 1 residue pairs ending at position *j *form the string *δ*·*b*. According to this definition, the residue pair at position *j *is *b*. The fp rate we want to compute is ∑_*θ*≥*T *_∑_*δ*·*b*∈*K *_*P*_*s*_(*θ*, *δ*·*b*), where *K *is the set of all the residue pair strings of length min(*k *+ 1, *s*).

It remains to compute *P*_*j*_(*θ*, *δ*·*b*) We first define *low *to be the smallest score in the matrix *M *and *high *to be the largest such score. Let *δ*·*b *be a residue pair string with length *k*' = min(*k *+ 1, *j*). If *j *∈ {*x*_1 _... *x*_*w*_} we have

Pj(θ,δ⋅b)=Pr⁡[b|δ]×∑b0∈A2Pj-1(θ−Μ[b],b0⋅δ).     (1)
 MathType@MTEF@5@5@+=feaafiart1ev1aaatCvAUfKttLearuWrP9MDH5MBPbIqV92AaeXatLxBI9gBamrtHrhAL1wy0L2yHvtyaeHbnfgDOvwBHrxAJfwnaebbnrfifHhDYfgasaacH8akY=wiFfYdH8Gipec8Eeeu0xXdbba9frFj0=OqFfea0dXdd9vqai=hGuQ8kuc9pgc9s8qqaq=dirpe0xb9q8qiLsFr0=vr0=vr0dc8meaabaqaciaacaGaaeqabaWaaeGaeaaakeaacqWGqbaudaWgaaWcbaGaemOAaOgabeaakiabcIcaOGGaciab=H7aXjabcYcaSiab=r7aKjabgwSixlabdkgaIjabcMcaPiabg2da9iGbccfaqjabckhaYjabcUfaBjabdkgaIjabcYha8jab=r7aKjabc2faDjabgEna0oaaqafabaacbiGae4huaa1aaSbaaSqaaiab+PgaQjab+1caTGqaaiab9fdaXaqabaaabaGaemOyai2aaSbaaWqaaiabicdaWaqabaWccqGHiiIZimaacqaFaeFqdaahaaadbeqaaiab9jdaYaaaaSqab0GaeyyeIuoakiabcIcaOiab=H7aXjab=jHiTiab=X5anHGaaiab7TfaBjab+jgaIjab91faDjabcYcaSiabdkgaInaaBaaaleaacqaIWaamaeqaaOGaeyyXICTae8hTdqMaeiykaKIaeiOla4IaaCzcaiaaxMaadaqadaqaaiabigdaXaGaayjkaiaawMcaaaaa@7214@

Otherwise,

Pj(θ,δ⋅b)=Pr⁡[b|δ]×∑b0∈A2Pj−1(θ,b0⋅δ).     (2)
 MathType@MTEF@5@5@+=feaafiart1ev1aaatCvAUfKttLearuWrP9MDH5MBPbIqV92AaeXatLxBI9gBamrtHrhAL1wy0L2yHvtyaeHbnfgDOvwBHrxAJfwnaebbnrfifHhDYfgasaacH8akY=wiFfYdH8Gipec8Eeeu0xXdbba9frFj0=OqFfea0dXdd9vqai=hGuQ8kuc9pgc9s8qqaq=dirpe0xb9q8qiLsFr0=vr0=vr0dc8meaabaqaciaacaGaaeqabaWaaeGaeaaakeaacqWGqbaudaWgaaWcbaGaemOAaOgabeaakiabcIcaOGGaciab=H7aXjabcYcaSiab=r7aKjabgwSixlabdkgaIjabcMcaPiabg2da9iGbccfaqjabckhaYjabcUfaBjabdkgaIjabcYha8jab=r7aKjabc2faDjabgEna0oaaqafabaWexLMBbXgBcf2CPn2qVrwzqf2zLnharCGvLjhzH5wyaGqbciaa+bfadaWgaaWcbaGaa4NAaiab=jHiTGqaaiab9fdaXaqabaaabaGaemOyai2aaSbaaWqaaiabicdaWaqabaWccqGHiiIZimaacqaFaeFqdaahaaadbeqaaiab9jdaYaaaaSqab0GaeyyeIuoakiabcIcaOiab=H7aXjabcYcaSiabdkgaInaaBaaaleaacqaIWaamaeqaaOGaeyyXICTae8hTdqMaeiykaKIaeiOla4IaaCzcaiaaxMaadaqadaqaaiabikdaYaGaayjkaiaawMcaaaaa@75C3@

The probability Pr[*b*|*δ*] is given by the model ℳ
 MathType@MTEF@5@5@+=feaafiart1ev1aaatCvAUfeBSjuyZL2yd9gzLbvyNv2CaerbwvMCKfMBHbqedmvETj2BSbWenfgDOvwBHrxAJfwnHbqeg0uy0HwzTfgDPnwy1aqee0evGueE0jxyaibaieYdOi=BH8vipeYdI8qiW7rqqrFfpeea0xe9Lq=Jc9vqaqpepm0xbbG8FasPYRqj0=yi0lXdbba9pGe9qqFf0dXdHuk9fr=xfr=xfrpiWZqaaeaabiGaaiaacaqabeaadaqacqaaaOqaaGWaaiab=ntinbaa@3AA6@. Equations (1) and (2) give the general case of the recurrence; for *j *<*k *+ 1, *δ*·*b *is a residue pair string of length *j*, and there is no prior residue 60 on the right-hand side. We initialize the recurrence by computing *P*_1_(*θ*, *b*) directly for all residue pairs *b *∈ A
 MathType@MTEF@5@5@+=feaafiart1ev1aaatCvAUfKttLearuWrP9MDH5MBPbIqV92AaeXatLxBI9gBamrtHrhAL1wy0L2yHvtyaeHbnfgDOvwBHrxAJfwnaebbnrfifHhDYfgasaacH8akY=wiFfYdH8Gipec8Eeeu0xXdbba9frFj0=OqFfea0dXdd9vqai=hGuQ8kuc9pgc9s8qqaq=dirpe0xb9q8qiLsFr0=vr0=vr0dc8meaabaqaciaacaGaaeqabaWaaeGaeaaakeaaimaacqWFaeFqaaa@3821@^2^.

We need to calculate *P*_*j*_(*θ*, *δ*·*b*) for 0 ≤ *j *≤ *s*. For each *j*, suppose the number of matching positions up to *j *is *π*(*j*). Then *θ *is bounded by the closed interval [*π*(*j*)·*iow*, *π*(*j*)·*high*]. Thus, the fp rate of the score seed with weight *w *and span *s *under a *k*th-order Markov model can be computed in time Θ(*w*(*high *- *low*)(|A
 MathType@MTEF@5@5@+=feaafiart1ev1aaatCvAUfKttLearuWrP9MDH5MBPbIqV92AaeXatLxBI9gBamrtHrhAL1wy0L2yHvtyaeHbnfgDOvwBHrxAJfwnaebbnrfifHhDYfgasaacH8akY=wiFfYdH8Gipec8Eeeu0xXdbba9frFj0=OqFfea0dXdd9vqai=hGuQ8kuc9pgc9s8qqaq=dirpe0xb9q8qiLsFr0=vr0=vr0dc8meaabaqaciaacaGaaeqabaWaaeGaeaaakeaaimaacqWFaeFqaaa@3821@|^2^)^*k*+1^*s*). The actual computational cost can be decreased substantially by observing that *P*_*j*_(*θ*, *δ*·*b*) is zero for many values of *θ*, since the different score values in score matrix *M *are not successive integers. In addition, for a specified threshold *T*, the lowest possible score sum *θ *at position *j *is *T *- (*w *- *π*(*j*))·*high*. Thus, we only need to compute *P*_*j*_(*θ*, *δ*·*b*) with *θ *bounded by the closed interval [max(*T *- (*w *- *π*(*j*))·*high*, *π*(*j*)·*iow*), *π*(*j*)·*high*].

#### Fp rate for multiple simultaneous seeds

We may compute the fp rate of a set of basic or transition seeds using the inclusion-exclusion method. Let Π = {*π*_1 _... *π*_*n*_} be a set of basic seeds or transition seeds. We are interested in the probability that *at least one seed *from Π yields a match. If more than one seed hits at the same position, only one hit is counted in the total number of seed hits. The inclusion-exclusion method is used to calculate the false positive rate *fp*_Π _as follows:

fpΠ=∑ifpπi−∑i,jfpπi+πj+∑i,j,kfpπi+πj+πk…     (3)
 MathType@MTEF@5@5@+=feaafiart1ev1aaatCvAUfKttLearuWrP9MDH5MBPbIqV92AaeXatLxBI9gBaebbnrfifHhDYfgasaacH8akY=wiFfYdH8Gipec8Eeeu0xXdbba9frFj0=OqFfea0dXdd9vqai=hGuQ8kuc9pgc9s8qqaq=dirpe0xb9q8qiLsFr0=vr0=vr0dc8meaabaqaciaacaGaaeqabaqabeGadaaakeaacqWGMbGzcqWGWbaCdaWgaaWcbaGaeuiOdafabeaakiabg2da9maaqafabaGaemOzayMaemiCaa3aaSbaaSqaaGGaciab=b8aWnaaBaaameaacqWGPbqAaeqaaaWcbeaaaeaacqWGPbqAaeqaniabggHiLdGccqGHsisldaaeqbqaaiabdAgaMjabdchaWnaaBaaaleaacqWFapaCdaWgaaadbaGaemyAaKgabeaaliabgUcaRiab=b8aWnaaBaaameaacqWGQbGAaeqaaaWcbeaaaeaacqWGPbqAcqGGSaalcqWGQbGAaeqaniabggHiLdGccqGHRaWkdaaeqbqaaiabdAgaMjabdchaWnaaBaaaleaacqWFapaCdaWgaaadbaGaemyAaKgabeaaliabgUcaRiab=b8aWnaaBaaameaacqWGQbGAaeqaaSGaey4kaSIae8hWda3aaSbaaWqaaiabdUgaRbqabaaaleqaaaqaaiabdMgaPjabcYcaSiabdQgaQjabcYcaSiabdUgaRbqab0GaeyyeIuoakiablAciljaaxMaacaWLjaWaaeWaaeaacqaIZaWmaiaawIcacaGLPaaaaaa@6940@

where *fp*_*π *_is the fp rate for seed *π*, and the combined seed *π*_*i *_+ *π*_*j *_matches at a given position if and only if both *π*_*i *_and *π*_*j *_match at that position. For basic seeds, *π*_*i *_+ *π*_*j *_is actually the union of all the inspected positions of *π*_*i *_and *π*_*j*_. For a transition seed *π*_*i *_= (*X*_*i*_, *Z*_*i*_) with span *s*_*i *_and a transition seed *π*_*j *_= (*X*_*j*_, *Z*_*j*_) with span *s*_*j*_, *π*_*i *_+ *π*_*j *_is a transition seed with span max(*s*_*i*_, *s*_*j*_). Each position *k*, 0 ≤ *k *≤ max(*s*_*i*_, *s*_*j*_), is a match position if *k *∈ *X*_*i *_or *k *∈ *X*_*j*_. Otherwise, it is a transition position if *k *∈ *Z*_*i *_or *k *∈ *Z*_*j*_. *k *is a "don't care" if neither of the previous two conditions is satisfied. For example, the union of two transition seeds ({0,1, 3,4, 8}, {2, 7}) and ({0,1, 2, 5}, {4}) is ({0,1, 2, 3,4, 5, 8}, {7}).

The inclusion-exclusion method can be combined with a hashing strategy to estimate the fp rate for multiple simultaneous seeds without using a background Markov model. However, the union of two or more transition seeds usually has a large weight (e.g., *w *= 18), which still incurs a large cost for direct counting. In this case, we can use the first term of Equation (3) to estimate the seed set's fp rate, since the remaining the terms are much smaller than it.

#### Comparison of predicted vs. actual fp rates

We used the predicted fp rate to design a score threshold for a score seed. In addition, in order to estimate the error introduced by only using the first term in Equation (3) when computing the actual fp rate for a set of transition seeds, we evaluate each other term in Equation (3) using the theory prediction. Thus, we need to quantify how well our model-based estimates predict score seeds and transition seeds' actual fp rate in real DNA. We randomly generated 100 samples of each type of transition seed with *w *+ *m *= 12 and 11 and then counted the seed matches on randomly chosen pairs of human and mouse genomic DNA sequences, with a typical length of 10 megabases. We compared the measured and estimated false positive rates for our sampled seeds on these pairs of sequences.

A similar comparison was conducted for score seeds under the score matrix *M*_*nc *_described below in the Results section. Since direct counting for a score seed may take hours to complete in our implementation, the error distribution for these seeds reflects only 10 random underlying basic seeds, rather than 100.

Table 1 illustrates the observed differences, measured in percentage of actual fp rate, between our actual and estimated fp rates for two types of transition seeds and score seeds. The estimated fp rates are typically within 20% of the actual rates. For score seeds, the difference was typically within 15%. In exchange for the stated limits on accuracy, we obtained a greater than 600× speedup for transition seeds over direct counting in the rate at which we could evaluate fp rates.

### Searching for an optimal set of transition seeds

As part of our comparative analysis, we wish to evaluate sets of simultaneous transition seeds. We therefore modified Mandala to find sets of such seeds with maximal sensitivity for a fixed weight. It has been shown that finding an optimal set of seeds is NP-hard [9]. For this reason, most existing works rely either on exhaustive search [4], local search techniques [6], approximations [10] or other heuristics [11,20] to find globally or locally optimal seeds. It is not practical to exhaustively search for an optimal transition seed with a big span. Thus we extend the local search strategy for basic seed design [6] to seek a locally optimal set of transition seeds with maximal span *s*_*max*_.

Let a transition seed *π*_*z *_= {{*x*_1 _... *x*_*w*_}, {*z*_1 _... *z*_*m*_}} be the current seed. The local neighborhood of *π*_*z *_is the set of all seeds π′z
 MathType@MTEF@5@5@+=feaafiart1ev1aaatCvAUfKttLearuWrP9MDH5MBPbIqV92AaeXatLxBI9gBaebbnrfifHhDYfgasaacH8akY=wiFfYdH8Gipec8Eeeu0xXdbba9frFj0=OqFfea0dXdd9vqai=hGuQ8kuc9pgc9s8qqaq=dirpe0xb9q8qiLsFr0=vr0=vr0dc8meaabaqaciaacaGaaeqabaqabeGadaaakeaaiiGacuWFapaCgaqbamaaBaaaleaacqWG6bGEaeqaaaaa@3025@ that differ from *π*_*z *_in exactly one of the choices *x*_1 _... *x*_*w *_or *z*_1 _... *z*_*m*_. Let *Y *= {0 ... *s*_*max *_- 1} - *π*_*z *_be the set of all "don't care" positions. To generate a seed π′z
 MathType@MTEF@5@5@+=feaafiart1ev1aaatCvAUfKttLearuWrP9MDH5MBPbIqV92AaeXatLxBI9gBaebbnrfifHhDYfgasaacH8akY=wiFfYdH8Gipec8Eeeu0xXdbba9frFj0=OqFfea0dXdd9vqai=hGuQ8kuc9pgc9s8qqaq=dirpe0xb9q8qiLsFr0=vr0=vr0dc8meaabaqaciaacaGaaeqabaqabeGadaaakeaaiiGacuWFapaCgaqbamaaBaaaleaacqWG6bGEaeqaaaaa@3025@, one of three operations is allowed: swapping some match position *x*_*i *_with some transition position *z*_*j*_, swapping some *x*_*i *_with some "don't care" position *y*_*k *_∈ *Y*, or swapping some *z*_*j *_with some *y*_*k*_. The only restriction is that position 0 cannot be a "don't care". Using this neighborhood structure and the probability calculations described above for transition seeds, we employ hill climbing with random restarts in seed space to find a near-optimal seed. To design a set of simultaneous transition seeds Π_*Z*_, we extend the neighborhood definition to encompass all sets Π′z
 MathType@MTEF@5@5@+=feaafiart1ev1aaatCvAUfKttLearuWrP9MDH5MBPbIqV92AaeXatLxBI9gBaebbnrfifHhDYfgasaacH8akY=wiFfYdH8Gipec8Eeeu0xXdbba9frFj0=OqFfea0dXdd9vqai=hGuQ8kuc9pgc9s8qqaq=dirpe0xb9q8qiLsFr0=vr0=vr0dc8meaabaqaciaacaGaaeqabaqabeGadaaakeaacuqHGoaugaqbamaaBaaaleaacqWG6bGEaeqaaaaa@2FDF@ in which one transition seed π′zi∈Π′z
 MathType@MTEF@5@5@+=feaafiart1ev1aaatCvAUfKttLearuWrP9MDH5MBPbIqV92AaeXatLxBI9gBaebbnrfifHhDYfgasaacH8akY=wiFfYdH8Gipec8Eeeu0xXdbba9frFj0=OqFfea0dXdd9vqai=hGuQ8kuc9pgc9s8qqaq=dirpe0xb9q8qiLsFr0=vr0=vr0dc8meaabaqaciaacaGaaeqabaqabeGadaaakeaaiiGacuWFapaCgaqbamaaBaaaleaacqWG6bGEcqWGPbqAaeqaaOGaeyicI4SafuiOdaLbauaadaWgaaWcbaGaemOEaOhabeaaaaa@3641@ differs from the corresponding *π*_*zi *_∈ Π_*Z *_in a single position.

### Construction of test alignment set

We need a set of alignments on which to evaluate seed sensitivity. Many previous papers [4,7,9,10,14] have derived alignments from abundant sources of coding DNA, such as ESTs. In this work, however, we also wish to investigate the ability of seeds to detect alignments in noncoding DNA, in particular in regions of long-range noncoding sequence orthology in eukaryotes. We therefore constructed a test set, based on alignments of two mammalian genomes, that is biased against inclusion of alignments involving coding DNA.

Ideally, existing seeded alignment tools should not be directly applied to extract alignments for our test set, since any seed used by these tools would have perfect sensitivity to these alignments and would therefore introduce a strong bias. For example, Brejova *et al*. observed that their direct use of BLASTP made all the alignments in their training set contain the default BLASTP seed [14]. However, direct application of the Smith-Waterman algorithm [21] to sample alignments from noncoding regions between a pair of large genomes is not computationally feasible. We therefore combine seed heuristics and dynamic programming such that rigorous, seed-free dynamic programming is only applied on small sequence blocks.

Our construction procedure is as follows. It takes as input a pair of genomes and produces a set of ungapped local alignments, or *high-scoring segment pairs *(HSPs).

1. Extract orthologous pairs of regions from the human and mouse genomes according to a provided synteny table from the UCSC Genome Browser [13,22].

2. Remove from these regions any DNA annotated as coding for protein, according to the Twinscan gene structure predictor [23] and the known genes. Also, remove low-complexity DNA and known interspersed repeats.

3. Apply a seeded alignment tool on every pair of orthologous regions to extract gapped local alignments between them. We use a Karlin-Altschul E-value of 1.0 to keep more potential HSPs.

4. From the gapped local alignments for each region pair, chain together a subset of alignments whose total score is maximized under the following constraints: 1) Between any two alignments in the subset, one alignment's starting indices in two sequences cannot be smaller than another alignment's ending indices. 2) All the alignments in the subset must have the same orientation. This subset can be obtained using, e.g., the longest increasing subsequence algorithm given in [24].

5. Create small blocks (200 K × 200 K) within each pair of regions that cover the optimal chain obtained in step 4. To avoid missing HSPs at block boundaries, adjacent blocks overlap slightly.

6. For every block created in step 5, apply an ungapped version of the SIM algorithm [25] to extract all HSPs in the block with E-value ≤ 0.001.

**Figure 1:**
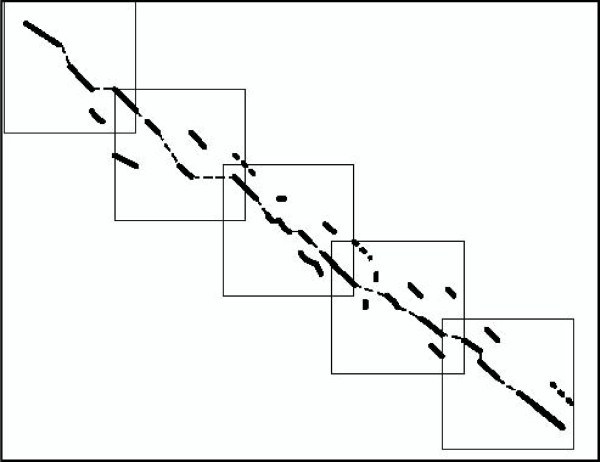
**Derivation of blocks in which to search for HSPs**. Blocks (dotted squares) are created along the maximum-weight chain (dashed thin lines) of gapped local alignments (thick black lines).

Fig. 1 illustrates a set of blocks created by the above procedure.

## Results

In this section, we compare the sensitivities and fp rates of different types of seed on sets of DNA sequence alignments. We work principally with our set of noncoding-biased alignments, described in the previous section, but we also measure performance on a set of EST-derived alignments, which has a stronger coding bias.

The experimental framework and data sources are described in detail in Appendix 1. All the seeds tested below were designed by the Mandala software using a Markov alignment model ℳ
 MathType@MTEF@5@5@+=feaafiart1ev1aaatCvAUfeBSjuyZL2yd9gzLbvyNv2CaerbwvMCKfMBHbqedmvETj2BSbWenfgDOvwBHrxAJfwnHbqeg0uy0HwzTfgDPnwy1aqee0evGueE0jxyaibaieYdOi=BH8vipeYdI8qiW7rqqrFfpeea0xe9Lq=Jc9vqaqpepm0xbbG8FasPYRqj0=yi0lXdbba9pGe9qqFf0dXdHuk9fr=xfr=xfrpiWZqaaeaabiGaaiaacaqabeaadaqacqaaaOqaaGWaaiab=ntinbaa@3AA6@ inferred from the test set. Although Mandala does not guarantee global optimality of the seeds it chooses, the seeds are locally near-optimal given their design constraints.

We illustrate the behaviors of different seed types using ROC curves to facilitate comparing the sensitivities of different seeds at the same fp rate (or vice versa). We report empirically measured sensitivities and fp rates for all seed types. However, as described above, we only used the first term of Equation (3) to compute the fp rates for transition seed sets, since a fully empirical rate computation proved computationally difficult. Fortunately, for the seed sets studied here, each component seed tends to inspect different groups of positions [8], and so their union has a much larger weight than any individual seed. Hence, the first term of Equation (3) indeed dominates. By estimating each term of Equation (3) using the dynamic programming method, we estimate that this simplification introduces at most 10% error in our fp rates for transition seed sets.

### Performance of transition seeds on noncoding test set

We first investigated how the transition weight *m *and the set size *n *affect the performance of transition seeds. According to our definitions of *w *and *m *for transition seeds, a transition position allows twice as many base pairs as a match position. Thus, the transition seeds with same value of *w*+*m*/2 have comparable specificity. We first designed experiments to investigate the sensitivity/specificity tradeoff by increasing *m *while keeping *w *+ *m*/2 unchanged. Secondly, we did experiments using multiple transition seeds.

**Figure 2:**
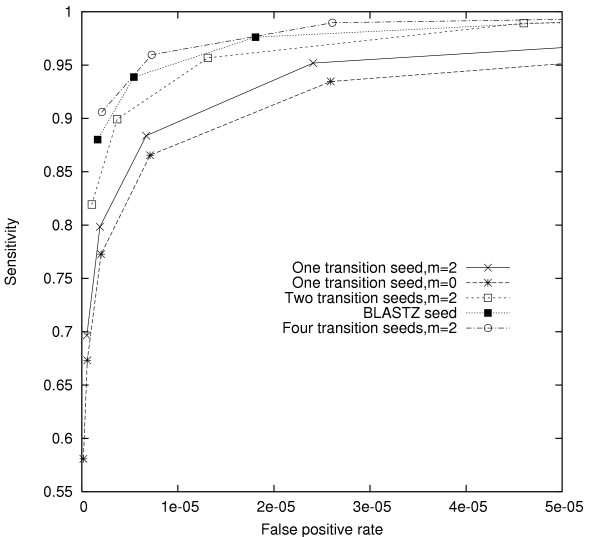
**Comparison of transition seeds designed for noncoding HSPs**. Each ROC curve shows seeds with the same transition weight *m *but different match weights *w*. The curve corresponding to BLASTZ seed starts with a seed with *w *= 11 and *m *= 1. All the other curves start with a seed with *w *+ *m*/2 = 11. For each curve, the subsequent points decrease *w *by 1 while holding *m *constant. Each point represents a locally optimal set of seeds.

Fig. 2 compares the performance of two types of single transition seeds with *m *= 0 (basic seeds) and *m *= 2, as well as two transition seeds, four transition seeds, and one BLASTZ seed. On our test set, increasing the number *n *of simultaneous transition seeds used significantly improves seed performance, with the ROC curves for larger *n *dominating those for smaller *n*. These results are consistent with those observed for basic seeds in previous work (e.g., [6]). Comparison between single transition seeds with *m *= 0 and *m *= 2 shows that increasing *m *for fixed *n *+ *m*/2 also improves seed performance.

The BLASTZ seed tested in Fig. 2 introduces only a single transition; however, the position of that transition is not fixed, in contrast to the other seeds tested in this experiment. Our results indicate that allowing such "freedom of movement" is highly effective: the single BLASTZ seed performed at least as well as a pair of seeds with fixed transitions, and its performance approaches that of four such seeds. Moreover, as we discuss below, there are algorithmic reasons to prefer the BLASTZ seed in practice.

### Performance of score seeds on noncoding test set

Recall that a score seed is a pair <*π*, *T*>, where *π *is a basic seed and *T *is a score threshold. The score of a residue pair comes from a score matrix *M*. In this section, we investigate the impact of the matrix *M *on the performance of a score seed and attempt to evaluate which properties of alignments this matrix should capture to optimize its sensitivity/specificity tradeoff.

#### Design of score matrices

In similarity search, the matrix *M *distinguishes the alignments being sought based on their unusual residue composition. We constructed matrices that captured three increasingly refined models of this composition, to quantify which signals were most important to score seed performance.

First, we designed our own symmetric score matrix *M*_*nc*_, based on the log-likelihood ratio methodology described in [26,27]. Suppose model ℳ
 MathType@MTEF@5@5@+=feaafiart1ev1aaatCvAUfeBSjuyZL2yd9gzLbvyNv2CaerbwvMCKfMBHbqedmvETj2BSbWenfgDOvwBHrxAJfwnHbqeg0uy0HwzTfgDPnwy1aqee0evGueE0jxyaibaieYdOi=BH8vipeYdI8qiW7rqqrFfpeea0xe9Lq=Jc9vqaqpepm0xbbG8FasPYRqj0=yi0lXdbba9pGe9qqFf0dXdHuk9fr=xfr=xfrpiWZqaaeaabiGaaiaacaqabeaadaqacqaaaOqaaGWaaiab=ntinbaa@3AA6@ describes the distribution of residue pairs for an alignment. For each ordered pair of residues *i *and *j*, Pr⁡ℳ(i,j)
 MathType@MTEF@5@5@+=feaafiart1ev1aaatCvAUfKttLearuWrP9MDH5MBPbIqV92AaeXatLxBI9gBamrtHrhAL1wy0L2yHvtyaeHbnfgDOvwBHrxAJfwnaebbnrfifHhDYfgasaacH8akY=wiFfYdH8Gipec8Eeeu0xXdbba9frFj0=OqFfea0dXdd9vqai=hGuQ8kuc9pgc9s8qqaq=dirpe0xb9q8qiLsFr0=vr0=vr0dc8meaabaqaciaacaGaaeqabaWaaeGaeaaakeaacyGGqbaucqGGYbGCdaWgaaWcbaacdaGae83mH0eabeaakiabcIcaOiabdMgaPjabcYcaSiabdQgaQjabcMcaPaaa@3FA6@ is the frequency of the pair (*i*,*j*) in model ℳ
 MathType@MTEF@5@5@+=feaafiart1ev1aaatCvAUfeBSjuyZL2yd9gzLbvyNv2CaerbwvMCKfMBHbqedmvETj2BSbWenfgDOvwBHrxAJfwnHbqeg0uy0HwzTfgDPnwy1aqee0evGueE0jxyaibaieYdOi=BH8vipeYdI8qiW7rqqrFfpeea0xe9Lq=Jc9vqaqpepm0xbbG8FasPYRqj0=yi0lXdbba9pGe9qqFf0dXdHuk9fr=xfr=xfrpiWZqaaeaabiGaaiaacaqabeaadaqacqaaaOqaaGWaaiab=ntinbaa@3AA6@. Let

Pℳ(i,j)=Pr⁡ℳ(i,j)+Pr⁡ℳ(j,i).     (4)
 MathType@MTEF@5@5@+=feaafiart1ev1aaatCvAUfKttLearuWrP9MDH5MBPbIqV92AaeXatLxBI9gBamrtHrhAL1wy0L2yHvtyaeHbnfgDOvwBHrxAJfwnaebbnrfifHhDYfgasaacH8akY=wiFfYdH8Gipec8Eeeu0xXdbba9frFj0=OqFfea0dXdd9vqai=hGuQ8kuc9pgc9s8qqaq=dirpe0xb9q8qiLsFr0=vr0=vr0dc8meaabaqaciaacaGaaeqabaWaaeGaeaaakeaaieGacqWFqbaudaWgaaWcbaacdaGae43mH0eabeaakiabcIcaOiabdMgaPjabcYcaSiabdQgaQjabcMcaPiabg2da9maaxababaGagiiuaaLaeiOCaihaleaacqGFZestaeqaaOGaeiikaGIaemyAaKMaeiilaWIaemOAaOMaeiykaKIaey4kaSYaaCbeaeaacyGGqbaucqGGYbGCaSqaaiab+ntinbqabaGccqGGOaakcqWGQbGAcqGGSaalcqWGPbqAcqGGPaqkcqGGUaGlcaWLjaGaaCzcamaabmaabaGaeGinaqdacaGLOaGaayzkaaaaaa@575E@

Suppose further that ℳ
 MathType@MTEF@5@5@+=feaafiart1ev1aaatCvAUfeBSjuyZL2yd9gzLbvyNv2CaerbwvMCKfMBHbqedmvETj2BSbWenfgDOvwBHrxAJfwnHbqeg0uy0HwzTfgDPnwy1aqee0evGueE0jxyaibaieYdOi=BH8vipeYdI8qiW7rqqrFfpeea0xe9Lq=Jc9vqaqpepm0xbbG8FasPYRqj0=yi0lXdbba9pGe9qqFf0dXdHuk9fr=xfr=xfrpiWZqaaeaabiGaaiaacaqabeaadaqacqaaaOqaaGWaaiab=ntinbaa@3AA6@_*b *_describes the distribution of pairs in a random alignment, and define

LLR(i,j)=log⁡[Pℳ(i,j)/Pℳb(i,j)].     (5)
 MathType@MTEF@5@5@+=feaafiart1ev1aaatCvAUfKttLearuWrP9MDH5MBPbIqV92AaeXatLxBI9gBamrtHrhAL1wy0L2yHvtyaeHbnfgDOvwBHrxAJfwnaebbnrfifHhDYfgasaacH8akY=wiFfYdH8Gipec8Eeeu0xXdbba9frFj0=OqFfea0dXdd9vqai=hGuQ8kuc9pgc9s8qqaq=dirpe0xb9q8qiLsFr0=vr0=vr0dc8meaabaqaciaacaGaaeqabaWaaeGaeaaakeaacqWGmbatcqWGmbatcqWGsbGucqGGOaakcqWGPbqAcqGGSaalcqWGQbGAcqGGPaqkcqGH9aqpcyGGSbaBcqGGVbWBcqGGNbWzcqGGBbWwcqWGqbaudaWgaaWcbaacdaGae83mH0eabeaakiabcIcaOiabdMgaPjabcYcaSiabdQgaQjabcMcaPiabc+caViabdcfaqnaaBaaaleaacqWFZestdaWgaaadbaacbiGae4NyaigabeaaaSqabaGccqGGOaakcqWGPbqAcqGGSaalcqWGQbGAcqGGPaqkcqGGDbqxcqGGUaGlcaWLjaGaaCzcamaabmaabaGaeGynaudacaGLOaGaayzkaaaaaa@5D7F@

A score matrix can be formed from the *LLR*(*i*,*j*) values by multiplying them by a suitably large constant and rounding the results to integers. Our matrix *M*_*nc *_was derived using probabilities estimated from our test alignments and the underlying sequences; it is given in Table 2.

We then designed two simplified matrices that used more limited information about the alignments to be found. Matrix *M*_0_, which follows the existing practice of NCBI BLASTN, uses only the overall probability of residue matches to recognize meaningful alignments; that is, all its diagonal entries are the same, as are all its off-diagonal entries. Matrix *M*_*z *_elaborates on *M*_0 _by assigning separate scores to matches, transitions, and transversions, but does not distinguish between all residue pairs as does the full *M*_*nc*_.

#### Impact of score matrices on score seed performance

We compared the performance, on our noncoding test set, of score seeds based on the matrices *M*_*nc*_, *M*_*z*_, and *M*_0_. We used a common, optimized basic seed *π *as the underlying seed for all score seeds with a given weight. Although *π *was not optimized individually based on different matrices, experiments showed that doing so did not improve performance vs. using a single common seed for all matrices.

The specificity of a score seed is determined in part by its score threshold. The thresholds for seeds under *M*_*z *_were set such that at most one transition and no transversion was allowed among the inspected positions; hence, these score seeds behave equivalently to BLASTZ seeds. The thresholds for seeds using *M*_0 _and *M*_*nc *_were set so as to obtain roughly comparable fp rates to the corresponding seeds using *M*_*z*_. These choices resulted in score seeds with practically useful fp rates.

**Figure 3:**
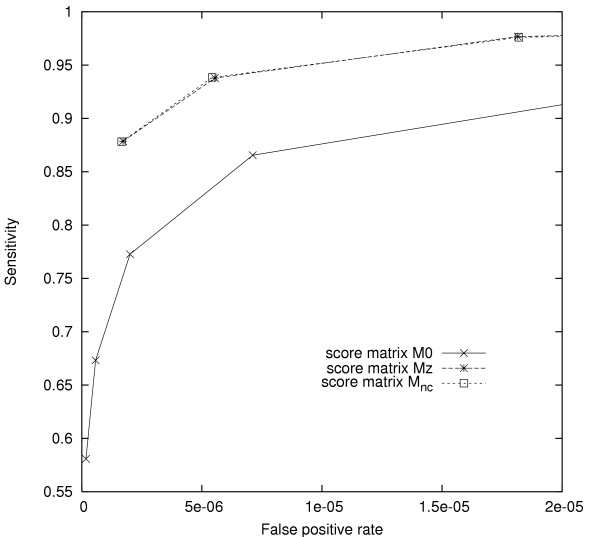
**Comparison of score seeds designed for noncoding HSPs**. Comparison of score seeds using different score matrices (*M*_0_, *M*_*z *_and *M*_*nc*_). The leftmost seed on each ROC curve has weight 12, while subsequent seeds decrease *w *by one. Score thresholds were chosen to produce similar fp rates for seeds of the same weight. Curves corresponding to score seeds under *M*_*nc *_and *M*_*z *_nearly coincide.

Fig. 3 gives the results of our comparison. Score seeds based on the matrix *M*_*z*_, which distinguishes transitions from transversions, dominated seeds based on *M*_0_, exhibiting superior sensitivity at comparable specificity. Because the underlying basic seeds were unchanged, the increase in performance is attributable to the extra information in matrix *M*_*z*_. In contrast, score seeds using the full matrix *M*_*nc *_exhibited performance indistinguishable from that of seeds using *M*_*z*_.

We infer that, with respect to our test set and choices of threshold, the extra information captured by the more complex *M*_*nc *_does not yield extra benefits for score seed design, vs. simply using the transition/transversion distinction encoded in *M*_*z*_. While we might observe different results with substantially less stringent score thresholds, these thresholds would likely result in fp rates too high for the resulting score seeds to be useful in practice.

### Comparison to coding-biased DNA – EST sequence alignments

Our results thus far have been obtained using a data set biased *against *alignments of coding sequences. To test whether these results apply to a data set with the opposite bias, we repeated the experiments of the last two sections on a set of alignments between human and mouse expressed sequence tags (EST). These alignments are by definition of transcribed DNA and contain a large proportion of coding DNA.

**Figure 4:**
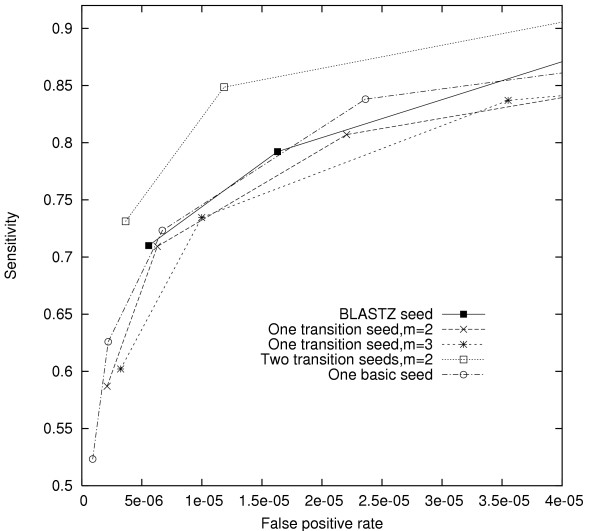
**Comparison of transition seeds on EST sequence alignment**. Each ROC curve shows seeds with the same transition weight *m *but different match weights *w*. Each curve starts with a seed with *w *+ *m *= 11 and then decreases *w *while holding *m *constant. Each point represents a locally optimal set of seeds. A ROC curve for single basic seeds is also plotted for a comparison.

**Figure 5:**
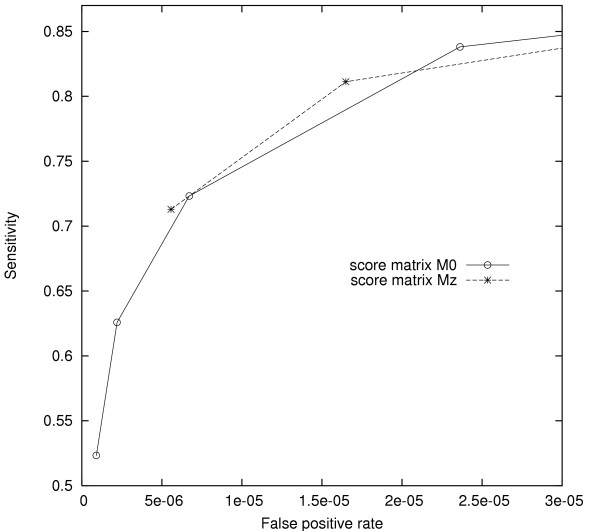
**Comparison of score seeds on EST sequence alignment**. Comparison of score seeds on EST sequence alignment using different score matrices (*M*_0_, *M*_*z*_). The leftmost seed on each ROC curve has weight 11, while subsequent seeds decrease *w *by one. Score thresholds were chosen to produce similar fp rates for seeds of the same weight.

Fig. 4 reports results from the comparison of basic, transition, and BLASTZ seeds, while Fig. 5 reports results for the comparison of score seeds. In contrast to our previous results, we found that BLASTZ and transition seeds conferred *no *benefit over the same number of basic seeds, and that the *M*_*z *_matrix did *not *improve on the simpler *M*_0 _matrix. Hence, our seeds appeared to behave qualitatively differently on coding DNA alignments.

To explain the different results for our two alignment sets, we must look to the statistical properties of their constituent HSPs. Firstly, EST alignments were better conserved overall (77%) than noncoding alignments (74%), blunting the advantage of any seed design that relies on information at non-matching positions. Second, among all non-matching positions in the EST alignments, 62% were transversions, vs. 38% transitions. The corresponding numbers for the noncoding test set were 40% transversions and 60% transitions. Hence, we expect that seed design strategies intended to exploit the higher frequency of transitions in conserved regions will fail for the EST alignment set, in which this signal is absent! These observations agree with and generalize those of [15] for a single transition seed.

### Computational costs of BLASTZ vs. transition seeds

The initial stage of seeded alignment search tools usually relies on a hashing strategy to detect all seed matches. For a basic seed, a query of size *L *(*L *≪ 4^*w *^for DNA) generates a hash table containing Θ(*L*) entries, corresponding to the sets of residues inspected by the seed at each position of the query. A search using *n *simultaneous basic seeds need *n *such hash tables. As *n *increases, the storage for these tables, as well as the cost of checking several tables when searching at each position of a database, may become prohibitively large.

A *neighborhood strategy *may be used to build hash tables for score seeds. For every offset in *L*, besides the original table entry extracted from the query, an entry is also created for each string that initiates a seed match with the query. All the strings that initiate a seed match with a given string constitute a *neighborhood *of that string. BLASTP's hash lookup stage provides one example of this well-known strategy. If the average neighborhood size for a single query offset is *k*, the number of entries in the hash table is Θ(*kL*). Dynamic programming can be used to estimate the average neighborhood size based on the weight *w *of a score seed, the score matrix *M*, and the score threshold *T*. BLASTZ seeds can be accommodated as a special case of this neighborhood strategy.

A neighborhood strategy can also be used to build hash tables for transition seeds. However, a more space- and time-efficient indexing method exists for seeds with transitions in fixed positions. By using an encoding of nucleotides for which A/T and C/G differ in the same bit position (e.g., A-00, C-01, G-10 and T-11), we can avoid neighborhood generation altogether for these seeds.

Table 3 compares the numbers and asymptotic sizes of hash tables for specific cases of each seed type with the best performance on our noncoding data set. For each seed type, we consider the hashing strategy for it as outlined above.

In a high-performance DNA similarity search application, much time is spent simply hashing the query. Profiling NCBI BLASTN during a search of the human genome against a moderately large (25 kb) query sequence shows that roughly half the search is spent finding seed matches. Hence, a performance comparison between a search using several hash tables and one using a single, denser table will favor the latter, provided one approach does not generate vastly more false positive seed matches than the other. In the case of one BLASTZ seed (one table) vs. four transition seeds (four tables), we found above that these two designs have roughly comparable false positive rates; hence, a comparison of computational cost will favor the BLASTZ seed.

## Discussion and Conclusion

Seed design is an important aspect of seeded alignment. A good seed should exhibit a good tradeoff between sensitivity and specificity on the sequence alignments of interest, as well as being compatible with an overall efficient search implementation. In this work, we have evaluated the relative performance of different types of seed – basic, transition, BLASTZ, and score – on sets of alignments biased both toward and against conserved coding DNA. Our results should help to guide designers of seeded alignment tools in navigating the large space of possible search heuristics.

We draw from our results several qualitative observations for designing seeds for use in genomic DNA comparisons.

1. To detect alignments in primarily noncoding regions, allowing transitions in a seed, rather than just matches and mismatches, significantly improves a seed's performance.

2. When transitions represent an important signal, allowing them to occur at any position of the seed, rather than fixing them, confers a substantial cost/sensitivity advantage.

3. Using a score seed with a matrix that distinguishes almost every base pair, such as *M*_*nc*_, appears to confer no benefit over a BLASTZ seed in finding noncoding alignments.

4. When transitions are *not *an important signal for recognizing alignments, the above results do not apply. In particular, alignments of ESTs, which are biased in favor of coding DNA, do not appear to exhibit this signal.

Overall, our work emphasizes the importance of designing a seeded alignment strategy to fit the search objective, using appropriate design tools and representative test sets of alignments. For DNA, knowing the relative frequencies of transitions and transversions is particularly important to the design process.

As part of this work, we have extended our Mandala seed design software to design transition and BLASTZ-style seeds. Table 4 gives optimized BLASTZ seeds for several common search weights as produced by our software.

Seeded alignment heuristics are important not only for database search applications, such as BLAST, but also for whole-genome and multiple sequence alignment tools. Currently, pairwise seeded alignment is used to construct anchors [2,3] around which to build larger or multiple alignments. Alternatively, one could use seed-like heuristics to recognize significant similarities in three or more sequences at once, using the results to build a more accurate set of anchors. Designing such heuristics is an interesting problem for future work.

## Authors' contributions

JB designed the original Mandala software, and both authors contributed significantly to its implementation. YS is responsible for the experiments and extensions described in this paper. All authors read and approved the final manuscript.

## Appendix 1: Experimental framework

We derived our noncoding-biased test set from alignments between the human and mouse genomes. Both genomes (builds hg16 and mm4), along with synteny and known gene tables, were downloaded from the UCSC Genome Browser [13,22]. Seed performance was compared on alignments between annotated synteny blocks in these genomes. We removed interspersed repeats, low complexity DNA, and Twinscan-annotated coding DNA from these regions before comparison. About 1.4 million ungapped HSPs (high-scoring segment pairs) were extracted by the procedure described in the Methods section. HSP lengths ranged from 26 to 1164 base pairs with an average of 83. Percent identities ranged from 50% to 100% with an average of 74%.

To obtain coding-biased alignments, we followed a procedure similar to that used in [9], using human and mouse EST sequences from NCBI GenBank. To maintain a tractable problem size, we used two sets of ESTs, released in March 2005, that contain all new or revised human and mouse EST sequences within 30 days before the release date. There are 16065 human EST sequences and 4228 mouse EST sequences in these two sets. We applied an ungapped version of the SIM program to derive all the ungapped alignments with a score threshold 16 between all pairs of human EST sequences and mouse EST sequences under a scoring system with +1/-1 for match/mismatch. About 2.5 million HSPs with average length 35 base pairs were derived, with an average identity of 77%.

From our HSPs on noncoding regions, we trained a first-order Markov alignment model ℳ
 MathType@MTEF@5@5@+=feaafiart1ev1aaatCvAUfeBSjuyZL2yd9gzLbvyNv2CaerbwvMCKfMBHbqedmvETj2BSbWenfgDOvwBHrxAJfwnHbqeg0uy0HwzTfgDPnwy1aqee0evGueE0jxyaibaieYdOi=BH8vipeYdI8qiW7rqqrFfpeea0xe9Lq=Jc9vqaqpepm0xbbG8FasPYRqj0=yi0lXdbba9pGe9qqFf0dXdHuk9fr=xfr=xfrpiWZqaaeaabiGaaiaacaqabeaadaqacqaaaOqaaGWaaiab=ntinbaa@3AA6@. For coding HSPs, it is more appropriate to use a nonstationary Markov model or HMM [7,14] so that the codon structure can be incorporated into the model. However, since our current seed design tool Mandala does not yet support nonstationary Markov models for transition seeds, we simply trained a third-order Markov model on EST alignments. The background model used to estimate fp rates was computed by combining two third-order Markov sequence models trained on the human and mouse genomes, respectively.

## Figures and Tables

**Table 1:** Comparison of estimated fp rates vs. the actual ones for score seeds and transition seeds. Entries show the distributions of the percent differences between estimated and actual fp rates for single score seeds with *w *= 11 and 12 and single transition seeds with *w *= 9 and 10 and *m *= 2.

	0–10%	10–15%	15–20%	20–25%
score seed w11	0.8	0.2	0	0
transi-nl-w9-m2	0.02	0.88	0.1	0
score seed w12	0	1	0	0
transi-n1-w1O-m2	0	0	0.8	0.2

**Table 2:** Score matrix *M*_*nc*_

	A	C	G	T
A	71	-108	-31	-125
C	-108	84	-109	-32
G	-31	-109	84	-108
T	-125	-32	-108	71

**Table 3:** Cost of hashing strategies with three classes of seed. *L*: size of query sequence; *w*: total seed weight. By using efficient coding, only one entry is needed at each offset for a single transition seed.

	1 BLASTZ seed	4 transition seeds with *m *= 2
# hash tables (lookups)	1	4
# entries per hash table	*wL*	*L*

**Table 4:** Locally optimal single BLASTZ seeds for noncoding HSPs. A transition is allowed in any one of the inspected positions listed for a seed.

**(m + w)**	**Seed**
12	{0,1,2,4,5,9,11,12,15,16,17,18}
11	{0,1,2,3,7,10,11,13,15,16,17}
10	{0,1,2,5,6,8,12,13,14,15}
9	{0,1,2,6,7,9,10,12,13}
